# The Effect of Leadership Styles and Relational Contracts on Compensation Effectiveness and Employee Performance

**DOI:** 10.3390/bs15091201

**Published:** 2025-09-03

**Authors:** Nela Rakic, Sladjana Barjaktarovic Rakocevic

**Affiliations:** Faculty of Organizational Sciences, University of Belgrade, 11000 Belgrade, Serbia; sladjana.barjaktarovic.rakocevic@fon.bg.ac.rs

**Keywords:** relational contract, leadership, employee performance, compensation system

## Abstract

This study examines how managerial leadership styles influence the perceived effectiveness of compensation systems and employee performance. While prior research on organizational control has focused on optimizing compensation structures, it often neglects the role of managers within these systems. Drawing on survey data from a large international bank in Serbia, the study finds that transformational leadership enhances employees’ perceptions of compensation system effectiveness. Furthermore, managers who rely more extensively on relational contracts foster greater intrinsic motivation and perceptions of fairness, thereby increasing system effectiveness. The study also reveals that managerial performance evaluations significantly affect employee productivity—but only when the compensation system is perceived as effective. This research contributes to the literature on leadership by highlighting the substantial impact of leadership styles on the use and outcomes of relational contracts within organizations.

## 1. Introduction

Compensation is a key element in the employee–organization relationship. Systems perceived as effective increase job satisfaction and motivation and steer behavior toward organizational goals (Lawler, 1990). Properly designed compensation systems impact strategic performance and outcomes (Eisenhardt, 1989; Holmstrom & Milgrom, 1994; Rousseau, 1989; Baker et al., 2002; Gibbons & Henderson, 2012). Most studies examine contingencies such as company size, competitive environment, or alignment with strategy (Balkin & Gomez-Mejia, 1990), but they largely ignore the human factor. Managers play a crucial role in shaping perceptions of compensation effectiveness through decisions on performance evaluations and bonus allocations. This study investigates how relational contracts and leadership styles affect employees’ perceptions of compensation systems and subsequent performance. It also examines the link between leadership style and psychological contracts, addressing a gap in research on how leaders influence these contracts (Oorschot et al., 2021).

The perceived effectiveness of a compensation system depends on whether managers cultivate relational contracts—trust-based agreements that extend beyond formal job descriptions (Rousseau, 1989; M. J. Gibbs et al., 2005). Psychological contracts are classified as relational or transactional, differing in focus, duration, stability, scope, and tangibility (Rousseau & McLean Parks, 1993). Relational contracts are emotional, intrinsic, and indefinite, while transactional contracts are economic, extrinsic, specific, and short-term. Rooted in social exchange theory, psychological contracts reflect perceptions of reciprocal obligations between employees and the organization (Rousseau, 1995). Employees perceive compensation as effective when managers recognize their work and link effort to growth opportunities, fostering relational contracts. Managers who disregard relational components operate exclusively through formal systems, thereby diminishing employees’ perceptions of their effectiveness. Relational contracts communicate expectations and rewards through trust, supportive communication, recognition, and personal support (Guest, 2004; Grimmer & Oddy, 2007), enhancing intrinsic motivation, fairness, and productivity. Effective employee performance, encompassing behaviors and job responsibilities, is critical for organizational success (Yolande, 2024; Attahiru, 2021).

This study also examines how middle managers’ leadership styles influence compensation effectiveness. Leadership styles shape managerial actions that affect employee behavior (Uhl-Bien, 2006). While top management designs systems, middle managers operate within these controls to achieve goals. Research distinguishes transactional and transformational leadership styles (Bass, 1990; Puni et al., 2020; Wen et al., 2019).^1^ Transactional leaders adhere to formal systems, whereas transformational leaders inspire teams and prioritize interpersonal relationships. Transformational leadership enhances the use of relational contracts, motivating employees and improving perceptions of compensation effectiveness. Transformational leadership also influences organizational commitment through perceptions of justice (Ariasa et al., 2024).

To test the above predictions, we conducted a survey. We collected data from 24 branches of a large international bank in Serbia. These branches all use the same formal compensation system where bonuses and evaluations are attached to targets. The response rate was 51.46%, corresponding to a sample size of 106 usable cases out of the 206 paper-based questionnaires distributed.

The results show a strong positive correlation between transformational leadership style and relational contracts. Middle managers characterized by a transformational leadership style will be more eager to use relational contracts and develop a long-term relationship with their employees. For transactional leadership, such an effect is not observed. Results further show a positive and statistically direct impact of relational contracts on compensation system effectiveness. Managers who are perceived as engaging in relational contracts are often associated with compensation systems that employees consider effective. Moreover, results indicate that these managers achieve enhanced perceived compensation effectiveness through the cultivation of intrinsic motivation among their employees. They also dedicate efforts to establishing fairness in outcomes by reinforcing the linkages between effort, job responsibilities, investments in intangible assets, and prospective future rewards.

Our supplemental analysis demonstrates that higher perceived compensation effectiveness, driven by relational contracting, has tangible economic benefits for the organization. Bank managers use a forced rating system, and when ratings are fair and objective, they increase employees’ motivation to invest effort (Berger et al., 2013). The effect of manager ratings on productivity is moderated by employees’ perception of compensation effectiveness. Consistent with this, our results show that when employees view the compensation system as effective, manager ratings more strongly influence positive changes in behavior, reducing absenteeism and increasing engagement compared to situations where compensation is perceived as ineffective.

This study contributes to the literature in several ways. First, using a relational contracting framework (Rousseau, 1989), we show how a uniform compensation system can be perceived differently depending on managerial practices. Prior work on relational contracting has focused on performance conditions for principals (Bouwens et al., 2020; Bol & Lill, 2015), buyer–supplier relationships (Grafton & Mundy, 2017), or how controls violating relational contracts harm outcomes (Gallani et al., 2019). Little is known about which managers engage in relational contracting, the outcomes of relational elements, and how these outcomes are achieved. We show that transformational leaders operate on the basis of relational contracts, making compensation systems appear more effective to employees.

Second, this study extends research on leadership styles. While prior studies examined leadership in relation to control system choices (Pearce & Sims, 2002; Abernethy et al., 2010), we demonstrate that leadership style influences outcomes even at lower organizational levels. Transformational leaders, compared to transactional leaders, are more effective at fostering relational elements in interactions with employees.

Finally, we contribute to the literature on managerial discretion. In sectors such as banking, performance is difficult to fully capture through objective measures (Campbell, 2012), requiring middle managers to align employees with organizational goals (M. J. Gibbs et al., 2005). Prior research highlights the value of discretion and trust (M. Gibbs et al., 2004; Bol & Lill, 2015) but does not examine how leadership styles, particularly at the middle-management level, shape a trusting environment under formal compensation systems (Rotemberg & Saloner, 1993).

The subsequent sections of this paper are organized as follows: The Literature Review, Research Hypotheses, and Conceptual Model section explains the mechanisms through which relational contracts can enhance perceived compensation effectiveness and how leadership can influence these contracts, drawing upon foundational works from the extensive literature on relational contracts. The Method section outlines the empirical survey design, while the Results section presents the outcomes of the hypothesis tests. Finally, the Implications and Limitations section concludes the paper by providing a synthesis of the findings, acknowledging the study’s limitations and suggesting avenues for future research.

## 2. Literature Review, Research Hypotheses, and Conceptual Model

### 2.1. Relational Contracts and Compensation System Effectiveness

Generally, better performance measures lead to more effective incentives (Levin, 2003), but subjectivity is often an inevitable factor because writing complete contracts is not always possible (Levin, 2003; M. Gibbs et al., 2004). Indeed, while being very informative for easier jobs (Campbell, 2012), performance measures for employees may need to be supplemented with discretion of direct managers who can contextualize employee performance. This is particularly important in a service industry such as banking, where formal contracts can only partially capture performance. A compensation system plays a crucial role in enhancing employee performance by addressing fundamental needs. Well-designed compensation—both financial and non-financial—fosters motivation, engagement, and improved performance, while inadequate compensation reduces commitment, satisfaction, and motivation. Fair compensation also strengthens organizational commitment and reduces turnover (Efendi & Safaria, 2025). Employees in demanding roles who are engaged through relational contracts generally experience higher career success and satisfaction, while those under transactional contracts report lower levels of both (Chang et al., 2025). A key question is whether relational contracts can enhance perceived compensation effectiveness even within formal compensation systems. This paper posits that relational contracts enhance acceptance and trust in formal systems, making compensation systems appear more effective when business unit managers actively apply relational contracting.

Relational contracts, by their very nature, entail a degree of subjectivity, which may render them less precise and consistent when compared to formal, transactional compensation systems that are explicitly designed to align employee behavior with organizational objectives. Hence, making greater use of relational contracting within a formal compensation system by deviating more from the formal system might have no effect (or have a negative effect) on its perceived effectiveness, as employees might put trust in the formal system. Yet, we argue that business unit managers who act as direct managers may have an advantage when making greater use of relational contracts. This suggests that such use can still enhance the perception a formal compensation system as effective.

Firstly, business unit managers are often better informed about the effort employees invested and the results achieved than managers higher in the hierarchy (Jensen & Meckling, 1992). Therefore, business unit managers might be capable of building long-term relationships with their employees based on mutual understandings (Gibbons & Henderson, 2012). For example, Grafton and Mundy (2017) argue that regular meetings and informal contracts contribute to a strong commitment to collective values and encourage desirable behaviors. Because relational contracts are inherently subjective, managers have greater flexibility to motivate employees and reward their contributions. This discretion enables managers to align compensation systems with organizational goals. Bol (2011) emphasizes that such managerial discretion over compensation can significantly influence employee productivity.

Secondly, prior studies have also argued that the socio-emotional elements of relational contracts, such as loyalty and support (Rousseau & McLean Parks, 1993; Coyle-Shapiro & Kessler, 2000; Shore & Tetrick, 1994), might be important factors for employee perception of compensation system effectiveness. A relational contracting style is typically linked to socio-emotional elements, including praise and support for intermediate achievements. Gibbons and Kaplan (2015) suggest that, under relational contracts, formal measures can serve as agreements among employees, supporting informal management. By fostering mutual understanding and shared interpretations of formal performance metrics, relational contracts address the limitations of formal agreements (Baker et al., 2002) and can enhance perceptions of compensation effectiveness, forming the basis for our first hypothesis.

**H1:**  *Relational contracts positively affect compensation system effectiveness.*

### 2.2. Relational Contracts, Compensation System Effectiveness, and Mediating Role of Fairness Outcome and Intrinsic Motivation

In addition to the direct effect of relational contracts, managers’ use of relational contracts is hypothesized to be positively associated with perceptions of fairness and intrinsic motivation, which, in turn, enhance employees’ perceptions of the compensation system’s effectiveness. The banking sector is unique, as top management frequently drives productivity through targets, while incentives encourage employees to exceed minimum performance requirements. However, a strong emphasis on incentives can undermine intrinsic motivation, consequently affecting productivity (Gachter et al., 2008; Ryan & Deci, 2000). A key feature of relational contracts is the shared understanding and mutual support between managers and employees, which enhances productivity. Both parties must clearly understand and accept all aspects of the contract. Since contracts are inherently incomplete (Rousseau, 2004), the manager–employee relationship is vital for building trust and giving context and meaning to employees’ work. Additionally, providing fair rewards for contributions that cannot be explicitly captured in formal agreements is crucial. Darrington and Howell (2011) emphasize that intrinsic motivation constitutes a key dimension of the economic relationship and can be strengthened through the quality of the manager–employee relationship. Managers who emphasize relational elements can inspire employees by clearly communicating expectations and providing meaningful work context, thereby promoting voluntary effort. High-quality relationships foster a sense of obligation, enabling employees to recognize their responsibilities without reliance on commands or financial incentives. This dynamic supports employees’ fundamental needs for competence and self-determination (Gagné et al., 2018; Ryan & Deci, 2019). Employees will likely feel appreciated by a manager who engages in relational contracts as such managers will probably reward their effort more adequately. It is also likely that employees will consider their job meaningful, exciting, and enjoyable. In a relational contract each employee is likely to be valued for unique contributions fostering mutual understanding on what is expected. Osterloh et al. (2002) and Fishbach and Woolley (2022) point out that solid personal relationships filled with mutual understanding are crucial for cooperation as they can further generate intrinsic motivation. Bird (2005) argues that a close personal relationship in relational contracts goes beyond contractual obligations and is characterized by friendship, reputation, interdependence, and altruism. This fosters committed and satisfied employees who are intrinsically motivated to exert effort, confident that their manager will recognize and reward it. Such intrinsic motivation can reinforce compensation effectiveness. If there is mutual understanding of how efforts are rewarded, employees feel they can contribute to organizational goals. This, in turn, can affect the perceived effectiveness of the compensation system.

The second mediator expected to positively affect employee perception of compensation system effectiveness is fairness outcome. Employees are significantly concerned about fairness (Fehr et al., 2007). During periods of instability, trust in leadership and perceptions of fairness are critical factors in fostering employees’ sense of belonging and affective commitment (González-Cánovas et al., 2024). In terms of the content of relational contracts, Herriot and Pemberton (1996) find that their three most important aspects are work environment, compensation, and fairness. Fairness perception, and in particular fairness about outcomes, leads to positive reactions from employees and positive attitudes towards their work (Lau & Oger, 2012). As such, outcome fairness leads to positive perceptions of compensation effectiveness.

In terms of the relationship between relational contracts and fairness outcome, it is important to recognize that relational contracts are not based only on explicit and objective accounting information. As previously mentioned, relational contracts are more focused on trust and informal communication, which introduces managers’ subjectivity in the evaluation process. Therefore, we suggest that the quality of the manager–employee relationship is crucial for employee perceptions of outcome fairness (Bianchi et al., 2015). Both managerial and employee behaviors play a critical role in addressing the limitations inherent in incomplete formal contracts (M. Gibbs et al., 2004). For a high perception of outcome fairness, it is very important that managers adequately reward employee effort, and that investments in training and education are recognized in the long-term performance evaluation (Rousseau, 2004). Perceptions of outcome fairness can be strengthened when employees feel they can rely on their managers in stressful situations, such as unmet targets or extended deadlines (Rousseau, 2004). Conversely, when relational contracts are less emphasized, employees may perceive that only short-term target achievement is valued, while efforts directed toward long-term objectives receive insufficient recognition. This imbalance can undermine perceptions of fairness, ultimately reducing the perceived effectiveness of the compensation system. Having all this in mind, we propose the following mediation hypotheses:

**H2a:**  *Relational contracts have a positive effect on intrinsic motivation and outcome fairness.*

**H2b:**  *The impact of relational contracts on compensation effectiveness is mediated by intrinsic motivation and outcome fairness.*

### 2.3. Leadership Styles as Antecedents of Relational Contracts

Leadership style significantly influences employee performance and trust in managers. Positive leadership fosters employee identification, support, and trust, which in turn encourages behaviors that benefit the organization (C. C. Lee et al., 2023). Olley (2021) highlights how leaders’ use of power and influence shapes team dynamics, offering insight into the impact of leadership on employee behavior and performance.

Social exchange theory stands as one of the most impactful conceptual frameworks for interpreting employee behavior within the workplace. A core tenet of this theory posits that workplace relationships develop over time into bonds characterized by mutual trust, loyalty, and commitment (Hutama et al., 2024).

Grounded in social exchange theory, employees who perceive equitable treatment from their organization are more inclined to reciprocate with enhanced performance and commitment (Hutama et al., 2024). Baker et al. (2002, p. 74) point out that “understanding the role of managers, who design and implement the relational contract that underpin informal organizational processes, is essential to understanding firms”. Osterloh et al. (2002) consider personal relationships as a precondition for establishing relational contracts. Relational contracts have a mediating role between organizational characteristics and the attitudes and behaviors of people who work for the organization (McDermott et al., 2013). Tabernero et al. (2009, p. 1392) suggest that relational contracts include “the exchange of socio-emotional resources”. This implies that the use of relational contracts strongly depends on managers and their leadership style. Leadership styles refer to the actions managers take to influence employee behavior (Uhl-Bien, 2006). These actions directly affect the use of relational contracts and signal whether managers adopt an open-ended, relational approach. Leadership effectiveness depends on the trust employees place in their managers (Song et al., 2024), which also encourages extra-role behaviors beyond formal obligations (Amir, 2019). The role of middle managers is particularly crucial in shaping perceptions of compensation system effectiveness. Managers who engage in relational contracting can build trust and more effectively motivate their workforce. Leadership style is also critical and may serve as a key factor for companies aiming to enhance the effectiveness of their compensation systems. To design and implement compensation systems optimally, HR departments should account for the quality of manager–employee relationships.

Leadership styles define actions that leaders undertake to align with the company strategy. There are several different definitions and categorizations of leadership styles, but the two most commonly contrasted—and referenced in this paper—are transactional and transformational. Transactional leadership is defined by the ways in which leaders may leverage employee compliance through the exchange of rewards (Alblooshi et al., 2021; Costa et al., 2023). Transactional leadership style is the most dominant style within the banking sector (Tuffour et al., 2022). However, it has been shown to contribute much less to employee commitment compared to a transformational leadership style. Transformational leaders create positive attitudes in employees and support a constructive workplace, while transactional leaders are less committed to doing so (McDermott et al., 2013). Transformational leaders pay close attention to each individual in the organization and strive to motivate employees toward organizational goals through personalized coaching and guidance (Bass, 1990; Steinmann et al., 2018; Krishnan, 2001). Transformational leadership encourages innovation by setting clear goals, inspiring employees, providing vision, and building trust. Leaders empower their teams and create a climate for innovation, leading to better results and a competitive advantage. By encouraging employees to think creatively, leaders inspire dedication and passion for their work (Pasamar et al., 2019; Alblooshi et al., 2021; Costa et al., 2023). Transformational leadership style enables managers and employees to share the company’s values and commit to the organization (Millward & Hopkins, 1998). Clear guidance helps employees understand expectations, positively contributing to the formation of a relational contract. Since transformational leadership style focuses on mutual needs, aspirations, and values, it has a long-term orientation; it requires time for cooperation between managers and employees (Yammarino et al., 1993). Syofyan et al. (2021) investigate how different leadership styles influence employees’ perceptions of evaluation fairness. The study finds that transformational leadership contributes to fairer evaluations by promoting open communication and trust. A manager’s role is crucial in developing relationships with employees; therefore, we argue that leadership style can affect the implementation of relational contracts. Unexpected fluctuations in productivity can challenge managers. Building strong manager–employee relationships is an effective strategy to manage these variations, as it boosts both individual performance and overall organizational productivity. Strong, empathetic leadership and constructive interactions create a resilient organizational framework (Al-Rabiey et al., 2024). Research indicates a positive correlation between transformational leadership and various elements, including three aspects of trustworthiness, confidence in leadership, and organizational citizenship behavior (J. K. Lee et al., 2024). Following the differences between leadership styles, we expect the transformational leadership style to positively influence relational contracts.

The effect of the transactional style on relational contracts is more difficult to predict. Although transactional leadership remains important in banking and can foster some shared understanding between employees and managers, research suggests that transactional leaders tend to exhibit lower trust in employee behavior, which may undermine relational contracting (McDermott et al., 2013; Raja et al., 2004). These leaders also focus on rules, targets, and obligations more strictly, but without clear guidance (laissez-fair approach). However, employees still find it difficult to understand what is expected of them. McInnis et al. (2009) further argue that commitment of employees is greater towards a transformational leader who views the contract as broad, trust-based, equal, negotiated, tangible, and long-term; such commitment arises less with a transactional leader. High distrust in managers practicing transactional leadership may even produce a negative effect (Raja et al., 2004). We therefore phrase the effect of transactional leaders on relational contracting in a null format. We summarize our discussion in the following hypotheses:

**H3a:**  *Transformational leadership style has a positive effect on the use of relational contracts.*

**H3b:**  *Transactional leadership style has no effect on the use of relational contracts.*

### 2.4. The Conceptual Model

**Figure 1:**
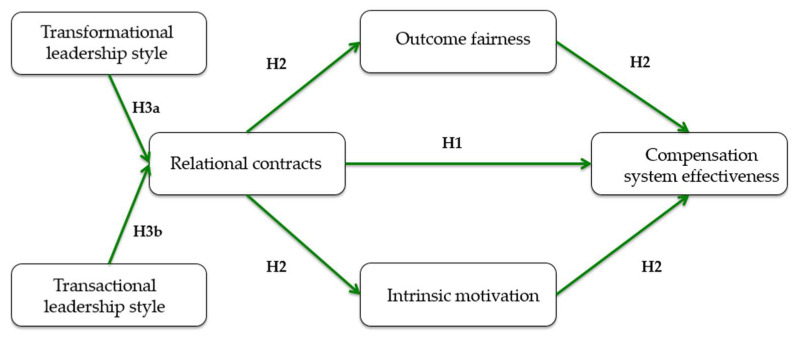
This figure presents the theoretical model and hypothesized relationships between variables. The model uses compensation system effectiveness as a dependent variable. Leadership styles (transformational and transactional) are used as antecedents of relational contracts. We predict that the effect of relational contracting on compensation effectiveness is mediated via the variables intrinsic motivation and fairness outcome. All constructs are defined in Appendix A.

The conceptual model developed in this study (see Figure 1) illustrates the proposed relationships between leadership styles, the use of relational contracts, and their effects on compensation system effectiveness. In summary, the model proposes that transformational and transactional leadership styles influence managers’ use of relational contracts. These relational contracts, in turn, improve employees’ sense of fairness and intrinsic motivation, which then positively impact their perception of how effective the compensation system is. The model is grounded in prior theoretical and empirical research on leadership behavior, psychological contracts, and compensation system design. Specifically, it draws upon social exchange theory and established findings suggesting that leadership styles shape how managers manage informal obligations and employee perceptions (Rousseau, 1989; Bass, 1990; Baker et al., 2002; M. J. Gibbs et al., 2005). These studies provide the theoretical foundation for the proposed pathways in the model.

## 3. Method

### 3.1. Sample Selection

Addressing the research questions requires the study of the perceived effectiveness of a compensation system at the business unit level. Data sets about compensation systems are often not publicly available, and formal compensation systems differ between organizations. Therefore, a survey has been developed to collect data from various branches of a large international bank in Serbia. All branches are subject to the same compensation system, where employees are evaluated on a set of similar targets.

Yet, branch managers still have discretion in assigning performance ratings to their employees. More precisely, at the end of each year managers are required to evaluate their employees and rate their performance (the final rating is based on three criteria: Job requirements, Operational Objectives, and Professional/Behavioral Development Objectives). Ratings range from 1 to 3 (1—Below the expectations; 2—Average performance; and 3—Above the expectations). In doing so, they should follow a pre-defined distribution (Berger et al., 2013; Chattopadhyay, 2019; Blume et al., 2009; Stewart et al., 2010; Grote, 2005). Specifically, bank managers should rate around 25% of employees below expectations, 50% in line with the expectations, and around 25% should receive an above the expectations rating.^2^ These ratings have been used in a subsequent analysis to study the effect of ratings on employee productivity. While a manager cannot directly influence the compensation system, ratings inform the final allocation of the bonus to an employee.^3^ This helps study variations in employee perception of their branch managers and the level of impact a particular branch manager with a certain leadership style has on the effectiveness of the formal compensation system at the branch.

Branch size was the main criterion for sample selection; branches had to have a minimum of five employees. Also, employees had to hold their current position for at least one year to guarantee familiarity with the compensation system. This means they had to experience at least one evaluation cycle to ensure that they could assess both their managers and the effectiveness of the system. The questionnaire was pretested with selected practitioners outside the bank to ensure the survey was understandable and to mitigate possible misinterpretation. All survey items were measured using a 5-point Likert scale to assess respondents’ agreement with the statements related to leadership styles, relational contracting, compensation effectiveness, and employee performance. The response options ranged from 1 (Strongly Disagree) to 5 (Strongly Agree). This scale format allowed participants to express varying degrees of agreement, providing nuanced data for analysis. Bank headquarters issued permission to conduct the survey. Branch managers were first informed about the survey, and one of the authors then visited each branch to distribute the questionnaire. Surveys were administered directly to the eligible employees (n = 206) at different branches. During the distribution we emphasized the importance of their participation and the confidentiality of their responses. The survey was supported by the top management and the HR department and promoted among the employees at different branches.^4^ The response rate was 51.45%, or a sample size of 106 usable cases out of the 206 paper-based questionnaires distributed at various branches. The sample included employees from 24 branches in five major cities in Serbia. Most branches were in Belgrade, but different municipalities were selected for a balanced sample size. As for the respondents’ demographics, the dataset comprises responses from 81 females and 25 males (n = 106), with ages ranging from 25 to 60, with a mean of 37.50 (SD = 6.70). As further shown in Table 1, work experience ranges from 1 to 30 years, with an average of 10.12 (SD = 5.05). The number of employees was taken as a branch size measure. The average branch has around 14.2 employees (SD = 8.13).^5^ Harman’s single factor test was employed to examine the vulnerability of the results to the common rater bias (Abernethy et al., 2017). A factor analysis was applied to all items associated with the dependent variable, yielding no indication that one factor accounts for the majority of covariance among the items. More precisely, the first factor accounted for 32.25% of the variance.

The survey measured employee perceptions of their manager’s leadership style, the extent of relational contracting, fairness perceptions, intrinsic motivation, and compensation system effectiveness, adapted from valid instruments in the literature. Appendix A provides the variable definitions of the constructs in our theoretical model. More precisely, Table A2 in Appendix A presents the demographic and organizational characteristics of the sample. This includes sex, age, work experience, branch size, and branch distribution across Serbian cities. As noted, participants were required to have held their current position for at least one year and work in a branch with five or more employees. In the section Variable Measurement below, we describe how constructs are being measured.

### 3.2. Variable Measurement

#### 3.2.1. Compensation System Effectiveness

Appendix A offers an overview of all the variables in the theoretical model. Gomez-Mejia and Welbourne (1988) define compensation systems as a set of compensation choices developed by managers that have the ability to affect organizational performance and employee behavior. Following Bedford et al. (2016), this research focuses on the measure of performance that should be related directly to the benefits derived from the choice of evaluation system and associated with the given strategic context. Compensation systems that are perceived as effective can be a crucial contributor to the achievement of goals and the motivation of employees (Balkin & Gomez-Mejia, 1990; Schuler & MacMillan, 1984). While pay policies are relatively similar, the perceived effectiveness as experienced by the employees may still vary depending on their direct manager. Our dependent variable—compensation system effectiveness—is measured using an instrument developed by Balkin and Gomez-Mejia (1990). As shown in Appendix A, six items refer to the extent to which the compensation system contributes to the achievement of organizational goals. The compensation system effectiveness scale has strong variation and a good internal consistency, with a Cronbach’s alpha coefficient of 0.86.

#### 3.2.2. Relational Contracts

The relational contracts scale was developed by retaining seven items from Grimmer and Oddy (2007) that should theoretically capture relational contracts. Grimmer and Oddy (2007) adopted the scale from Millward and Hopkins (1998). Our relational contracts scale has a good internal consistency, with a Cronbach’s alpha coefficient of 0.78. The scale contains questions about different relational contracting styles that can be used in a company. A relational contract motivates secure and stable relationships. Such contracts inspire individuals to engage at work and take greater levels of responsibility (Chen & Aryee, 2007). Relational contracts often rely on mutual understanding and reciprocity of effort, motivating employees to get the job done (Rousseau, 2004). Nevertheless, Gibbons and Henderson (2012) argue that employees may have expectations in terms of rewards and recognition, hoping that these are rewarded via development, growth, and promotion in an environment that relies on relational contracts. Therefore, items were selected carefully that focus not solely on the psychological definition of relational contracts (i.e., being part of a team, involvement), but also items that refer to reciprocity, growth opportunities, and recognition (i.e., promotion, growth, reciprocal exchange, etc.). The scale and various items relating to relational contracts have been validated in numerous studies, suggesting that they inspire workforce motivation (Gibbons & Henderson, 2012). The study investigated if branches where managers are perceived to make more use of relational contracts have higher perceived compensation effectiveness (as our key dependent variable).

#### 3.2.3. Fairness Outcome

Fairness outcome was measured using items from Lau and Oger (2012). The items we used refer to how much job descriptions explain rewards, whereby a better manager would tie rewards more towards job responsibilities. A branch manager who is able to do this is perceived to achieve higher outcome fairness (Lau & Oger, 2012). This study analyzes only fairness outcome because banking sector procedures are highly standardized, making it difficult to analyze procedural fairness. Also, having in mind that compensation system procedures are developed by the HR department and that the aim of this study is to analyze relationships between managers and employees in a branch, we focused only on aspects that are under a branch manager’s control. Managers have some control over how strongly rewards or outcomes are related to job characteristics (e.g., managers can suggest top priorities; they can decide to give a bonus to employees even if targets are not reached) and it is expected that managers who make more use of relational contracts achieve higher compensation effectiveness by stimulating more fairness outcome. Hartmann et al. (2010) indicate that fairness perceptions are important in explaining the relationship between accounting control systems and employee behaviors. The fairness outcome scale has a good internal consistency, with a Cronbach’s alpha coefficient of 0.87.

#### 3.2.4. Intrinsic Motivation

Intrinsic motivation was tested using the scale developed by Kuvaas et al. (2017). Their original scale, however, measures both intrinsic and extrinsic motivation. At branches where the manager stimulates relational contracts, it was expected that such contracts stimulate the intrinsic motivation of employees to work harder. The Cronbach’s alpha value for the intrinsic motivation scale exceeds the established empirical thresholds, resulting in a value of 0.86.

#### 3.2.5. Leadership Styles

Employee perception of a manager’s leadership style was measured using the Multilevel Leadership Questionnaire developed by Avolio and Bass (1995). Although the original scale was formulated for managerial use, this study involved employee participation in completing the questionnaire. The items thus measure how employees perceive their direct superior (Den Hartog et al., 1997). These perceptions are presumed to correlate with the actual leadership style of the manager (Bernhard & O’Driscoll, 2011; Vigoda-Gadot, 2007; Failla & Stichler, 2008).

The initial questionnaire comprised 21 items assessing 7 distinct leadership styles, which were subsequently categorized into two broader classifications: transformational and transactional (Avolio & Bass, 1988; Den Hartog et al., 1997). Items were divided into two factors after a factor analysis (PCA) of the 21 items with orthogonal rotation. Two factors are interpretable and contain enough items (eigenvalues for both factors are above 1). Additionally, these two factors explain 62.38% of variance. The transformational leadership scale contains 17 items relating to inspirational motivation, idealized influence, individual consideration, etc., as defined by the MLQ (Multifactor Leadership Questionnaire). The four items that load on transactional leadership style are more related to the laissez-fair approach or management by exception. Both scales have high internal consistency, with a Cronbach’s alpha coefficient of 0.96 for the transformational leadership style and 0.71 for the transactional leadership style.

#### 3.2.6. Control Variables

A series of control variables were employed in our model. Since compensation system effectiveness was a dependent variable, perceived long-term orientation and decentralization in terms of payment policy were used as control variables. Both control variables allow accounting for specific compensation-related information that can affect compensation effectiveness. Prior research further suggests that perceptions of employee commitment to a contract can rise when contracts are more long-term oriented and broader—captured by the long-term orientation—and feel as less imposed on employees (McInnis et al., 2009), which were captured via decentralization. The constructs are not included in Appendix A, but the items are listed in the explanatory footnote of Table 1. The long-term orientation scale refers to employee perception of whether the contract also includes long-term goals (Balkin & Gomez-Mejia, 1990). Internal consistency is rather low, with a Cronbach’s alpha coefficient of 0.48. The pay decentralization scale refers to the extent to which branch managers have the freedom to make compensation policy decisions and consequently the level of delegation they have within the existing pay policies (Balkin & Gomez-Mejia, 1990). The pay decentralization scale has a good internal consistency, with a Cronbach’s alpha coefficient of 0.64.

The number of employees was used as an indicator for the size of the branch. This number reflects the resource levels of the bank (Camanho & Dyson, 1999). Employees are the most valuable resource in service industries, so they are used as the most appropriate measure of branch size, which can affect perceptions of compensation effectiveness. A low number of employees can also indirectly affect the perceptions of compensation effectiveness as workload might be higher in such branches. We also controlled for sex as perceptions can differ between males and females. Finally, work experience in the banking industry was measured because Raja et al. (2004) suggest that contract dynamics change when being longer in the organization. As such, these dynamics might influence compensation system effectiveness.

### 3.3. Methodological Approach of Hypotheses Testing

Two approaches were used to test our hypotheses. First, a set of multiple linear regressions were used referring to the different paths (representing the hypotheses) of our theoretical model. Second, structural equation modelling was utilized to test the complete theoretical model. Multiple linear regressions were considered as appropriate to test all our hypothesis. IBM SPSS Statistics version 20 was used to analyze the dataset.

All hypotheses were tested using the same control variables (Baron & Kenny, 1986; Luft & Shields, 2003). Additionally, the full theoretical model was tested using a maximum likelihood method for structural equation model estimation—SEM (Schreiber et al., 2006). The path coefficients indicate the strength and direction of the relationship among the variables.

## 4. Results

### 4.1. Descriptive Statistics

Summary statistics displaying means and standard deviations are given in Table 1. Cronbach’s alpha was calculated for each of the measures derived from the survey (Cortina, 1993). As shown in Table 1, the constructs of the theoretical model have considerable variability. Employees who completed the survey had on average 10.12 years of experience, and the majority of the respondents were female (76%).

Table 2 provides the correlations among all variables. The study identified a strong positive correlation between relational contracts and intrinsic motivation (r = 0.49, *p* < 0.01), as well as between relational contracts and fairness outcome (r = 0.52, *p* < 0.01). The results further showed that both intrinsic motivation and fairness outcome positively correlated with compensation effectiveness (r = 0.53, *p* < 0.01; r = 0.39, *p* < 0.01). Additionally, the study identified a strong positive correlation between transformational leadership style and relational contracts (r = 0.49, *p* < 0.01). Therefore, managers with a transformational leadership style are more likely to use relational contracts. There is no correlation for the transactional leadership style and relational contracts (r = −0.07, *p* = 0.49). Generally, correlations are not high enough to warrant concerns about multicollinearity. The VIF of all subsequent models does not indicate any multi-collinearity concerns (Highest VIF < 2).

### 4.2. Hypothesis Tests

Before the assessment of the structural model, several regressions were run. The direct relationship between relational contracts and compensation system effectiveness was tested. As displayed in Table 3 (Model 1), regression coefficients show a significantly positive impact of relational contracts on compensation system effectiveness. Additionally, the results suggested that the examined attributes (relational contracts and control variables) explain 31% (R2 = 0.31; F6;99 = 7.58; *p* < 0.001) of variance of the dependent variable—perceived compensation system effectiveness. This therefore supports Hypothesis 1. More precisely, managers that are perceived to make more use of relational contracts with their employees achieve higher perceptions of compensation effectiveness.

According to our theoretical model, managers that are perceived to engage more in relational contracting will realize higher compensation effectiveness via outcome fairness and higher intrinsic motivation. Consistent with H2a, we first tested the first step in the mediation and explored whether relational contracts affect the mediator variables outcome fairness and intrinsic motivation. Table 3 shows that there is a positive and statistically direct impact of relational contracts on fairness outcome (Model 2) and intrinsic motivation (Model 3), consistent with the idea that relational contracting affects these two relationships.

As expressed in hypothesis H2b, we then ran regression using compensation system effectiveness as a dependent variable, and relational contracts, fairness outcome, and intrinsic motivation as independent variables. The idea is to explore if the impact of relational contracts flows through the mediators that we consider (Baron & Kenny, 1986). Table 4 reports the results of this analysis. The model has a strong explanatory power of 41% (R2 = 0.41; F8;97 = 8.39; *p* < 0.001). The mediator intrinsic motivation has a significant effect on the dependent variable (*p* < 0.01). The mediator fairness outcome is also positively associated with perceived compensation system effectiveness, but it is not significant at conventional levels of significance (*p* = 0.11).

Consistent with the criteria of Baron and Kenny (1986), while being significant in Model 1 of Table 2 (*p* < 0.01), the effect of relational contracts on compensation effectiveness reduces to non-significance in Table 4. This suggests that the effect of relational contracting is mediated by intrinsic motivation and fairness outcomes. Thus, branch managers that realize higher effectiveness of compensation systems in their organization through adopting a relational contract perspective do so by stimulating more intrinsic motivation among their employees. Also, outcome fairness seems to play a role (albeit less important than intrinsic motivation) in explaining the relation between relational contracts and compensation effectiveness. These results thus offer support for mediation (H2a/H2b).

As a final step, the characteristics of leaders who tend to engage more in relational contracts were explored. Two regressions were run, with relational contracts as the dependent variable and two leadership styles as independent variables, which allow us to test H3a and H3b. Regression coefficients are presented in Table 5. The results suggest that the transformational leadership style explains 29% (R2 = 0.29; F6;99 = 7.78; *p* < 0.001) of variance of the dependent variable relational contracts and is significant at the 1% level (*p* < 0.01). These results offer support for H3a, suggesting that transformational leaders engage more in relational contracting. For leaders that are more transactional, the relationship is less clear cut; they may even make less use of relational contracting. Results in Table 3 do not reveal a statistically significant impact of the transactional leadership style on relational contracts. Therefore, we cannot reject the null hypothesis H3b, which suggests that transactional leaders are likely not associated with higher or lower use of relational contracts.

#### Full Test of the Theoretical Model Using Structural Equation Model

**Figure 2:**
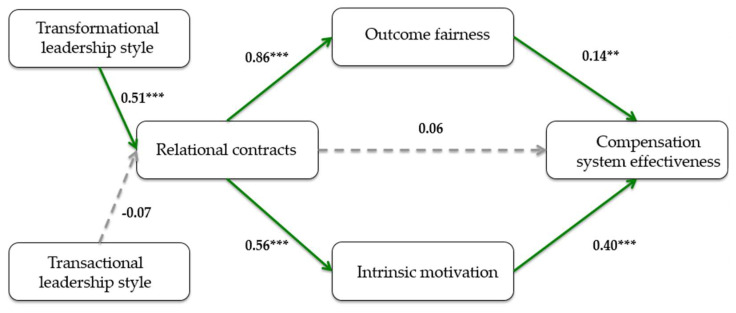
This figure presents the results of a theoretical model that uses compensation system effectiveness as a dependent variable and leadership styles (transformational and transactional) as antecedents of relational contracts (key independent variable). Intrinsic motivation and fairness outcome are the mediators. All constructs are defined in Appendix A. This analysis uses path analysis for the sample (n = 106). All paths displayed in this figure are estimated, and they are estimated jointly using a maximum likelihood method for structural equation model estimation—SEM (Schreiber et al., 2006). The standardized path coefficient and corresponding significance levels of the coefficients are shown next to each path. The paths with coefficients significant at the 0.10 level or less are depicted in solid lines, and other paths are in dotted lines. Goodness-of-fit of this model was calculated using the conventional Chi-square test, the Tucker–Lewis index, and the incremental fit index (Kline, 1998). All values are considered to be favorable. *** and ** denote significance at the 0.01 and 0.05levels, two-tailed.

Finally, the model with all variables was tested using structural equation model estimation. Figure 2 reports the theoretical model that uses compensation system effectiveness as the key dependent variable. Relational contracts is the key independent variable, and intrinsic motivation and fairness outcome are the mediators through which relational contracting should have an effect. The antecedents of relational contracting are the two leadership styles, namely transformational versus transactional. The sample size of 106 observations is sufficient for carrying out path analysis, given the degrees of freedom in our model (Kim, 2005). Generally, the model is adequate for the data. A conventional Chi-square test indicates that the covariance matrix implied by the model does not differ from the observed covariance matrix (χ^2^ = 11.57, *p* = 0.17). The Tucker–Lewis index and the incremental fit index, which show the proportion of improvement of the fit of the model over a null model, have values of 0.95 and 0.97, both above the acceptable level of 0.90 (Kline, 1998).

The path coefficients for our model are presented in Figure 2. All paths have significant coefficients, except the path from transactional leadership style to relational contracts and the path from relational contracts to perceived compensation system effectiveness. The path from transactional leadership style to relational contracts confirms the results of Table 5 earlier with regard to H3b. Since the path from relational contracts to compensation system effectiveness is not significant but relational contracts affect the mediators, which in turn affect perceived compensation effectiveness (Baron & Kenny, 1986), it can be concluded that relational contracts have an effect on perceived effectiveness of compensation via the mediator variables fairness outcomes and intrinsic motivation.

Overall, the leaders who are seen as more transformational are also perceived to make greater use of relational contracts. Relational contracts have an effect on the perceived compensation system effectiveness by stimulating fairness outcomes and intrinsic motivation. Our results thus stress the importance of the human element (leadership style and relational contracts), regarding an effective compensation system. The following section further explores if compensation effectiveness translates into real effects for the employees at the bank.

### 4.3. Supplemental Analyses

Employee performance refers to the actions and behaviors aimed at achieving set objectives, influenced by factors like proficiency, expertise, tenure, and commitment (Hutama et al., 2024). Managers rate employees on a 1 to 3 scale, with bonuses indirectly tied to these ratings. Ideally (i.e., when done fairly and objectively), such ratings should translate into higher motivation to expend effort at work (Berger et al., 2013). In Table 6 we examine the effects of ratings by managers on employee productivity. As argued in the literature, employee perceptions of the effectiveness of compensation policies can contribute to performance outcomes (Balkin & Gomez-Mejia, 1990; Schuler & MacMillan, 1984).

Our expectation is that compensation effectiveness acts as a moderator in the relationship between manager’s rating behavior and employee productivity. We predict that when compensation systems are perceived to be effective, ratings of managers have greater impact on employee productivity compared to when compensation policies are not perceived to be effective. That is, when compensation policies are perceived to be effective (as influenced by relational contracting elements), employees will perceive the ratings to be informative, increasing their motivation to work. Indeed, prior literature suggests that engagement at work can be a function of relational contracting elements, and given that relational contracts enhance compensation effectiveness (Chan, 2021; Braganza et al., 2021; Agarwal & Gupta, 2018; Guest, 2004), employees are more likely to trust the ratings of managers such that ratings are likely to trigger “real” changes in behavior when people perceive the system as effective.

Non-working hours was used as our key indicator for measuring productivity at the employee level. It represents engagement at work, which can be positively correlated with organizational outcomes (Bakker & Demerouti, 2007). More precisely, we use the number of non-working hours that is individually registered at the employee level during the year 2016, with an average of 23.15 (SD = 17.93), representing the numbers of hours the employee was not at work due to some personal issues (e.g., illness). We also have the performance ratings for each employee for that respective year as determined by their branch manager. Due to data limitations (for some employees, information on non-working hours or performance ratings are missing), we run the final sample with n = 83 employees. Other potentially relevant performance outcomes (such as revenue, etc.) are not made available to us by the bank, and are often also not collected at the individual employee level but determined by branch. Table 6 represents the regression results, with non-working hours as our key performance outcome. The same controls were used as in all previous analyses.

To begin, panel A of Table 6 assesses whether performance ratings provide meaningful information. The results in column (1) show that higher ratings reduce the number of non-working hours (t = −1.66, *p* = 0.10), meaning that ratings positively affect employee outcomes. In column two of Panel A, it has been shown that this negative relationship is stronger if compensation effectiveness is higher, as displayed by the interaction of ratings × compensation effectiveness, which is significant at the 10% level (t = −1.81, *p* = 0.07). Consistent with our prediction, the ratings that managers assign to their employees have a stronger effect on employee performance when compensation effectiveness is higher.

In panel B, we perform subsample analyses for the sample where perceived effectiveness is either low or high based on a median split on compensation effectiveness. Note that these results have to be interpreted with caution given that the sample size is relatively low in particular for the subsample of low effectiveness. Consistent with our conjecture above, column (1) of Panel B shows that ratings are not related to employee outcomes when compensation effectiveness is low (*p* = 0.73). However, ratings significantly reduce non-working hours when compensation effectiveness is high (t = −1.79, *p* = 0.08). This confirms that when compensation policies are perceived to be effective, ratings have a positive effect on performance outcomes (as measured by the reduction in non-working hours). These results do show that compensation effectiveness has real effects. That is, ratings matter much more in realizing better performance outcomes when employees perceive the compensation system to be effective. Thus, leaders that can manage this better will be desirable to the organization. That is, the antecedents and the mediators showed earlier influence effectiveness, which in turn has economic implications to the organization as it affects productivity at work.

## 5. Implications and Limitations

### 5.1. Theoretical Implications

The effectiveness of compensation systems is critical for organizational performance, employee satisfaction, and motivation to exert additional effort (Balkin & Gomez-Mejia, 1990; McDermott et al., 2013; Ryan & Deci, 2019; Fishbach & Woolley, 2022). Although prior literature in accounting has emphasized issues such as performance evaluation, target setting, and discretion in bonuses (Gupta & Shaw, 2014; Bol & Lill, 2015; M. Gibbs et al., 2004), relatively little attention has been given to understanding what makes employees perceive these systems as effective. Much of the existing work assumes that well-designed systems automatically improve outcomes, focusing primarily on structural and technical aspects. This study, however, shifts the focus to the human side by exploring how middle managers’ leadership styles shape employee perceptions of compensation system effectiveness. Specifically, it finds that managers who apply relational contracting—using trust, reciprocity, and relationship-building—are better able to enhance organizational outcomes even under rigid, predetermined compensation systems. This insight contributes to research on psychological contracts (Rousseau & McLean Parks, 1993), showing that employees’ experiences of fairness and motivation are deeply influenced by leadership behaviors. As a result, compensation effectiveness is not only a matter of system design but also of how managers foster trust and relational alignment with employees, which ultimately drives both satisfaction and performance.

The SEM analysis shows that transformational leaders are perceived as making greater use of relational contracts. Transformational behaviors—individualized support and trust-building—are directly linked to these trust-based agreements. Relational contracts significantly increase perceptions of compensation system effectiveness through two mechanisms: fairness and intrinsic motivation. Employees who experience fair and supportive leadership view compensation systems as just and motivating, which strengthens engagement and satisfaction. Both mediators have statistically significant effects, highlighting the need for leadership development that fosters transformational behaviors. Beyond compensation design, manager implementation and communication through relational practices are critical. Consistent with prior research (Balkin & Gomez-Mejia, 1990; McDermott et al., 2013; Ryan & Deci, 2019; Fishbach & Woolley, 2022), additional analyses confirm that effective compensation systems enhance job engagement and productivity.

This study responds to calls for a more comprehensive model of compensation systems (Murphy, 2013; Wall, 2020) by shifting focus from design elements to the human element in contracting. It shows that compensation practices are perceived as more effective when managers engage in relational contracts. Such managers build stronger relationships with employees by fostering intrinsic motivation and fairness, which in turn drive engagement. Balkin and Gomez-Mejia (1990) identify linking compensation systems to organizational strategies as a major challenge. Relational contracts provide a way to achieve this link, emphasizing the role of managers rather than system design alone. Our findings extend prior work on leadership style (Abernethy et al., 2010; Hartmann et al., 2010) by showing that middle managers, despite lacking authority to change formal systems, can shape employee effort through their leadership. Drawing on research on leadership styles (Tabernero et al., 2009; Uhl-Bien, 2006; McDermott et al., 2013; Osterloh et al., 2002) and psychological contracts (Raja et al., 2004), the study shows that transformational leadership fosters relational contracts, while transactional leadership has no significant effect in the banking sector. Transactional principles of structured tasks and rewards do not build the trust-based agreements that relational contracts require. Thus, leadership style matters not only for designing controls, as prior research suggests, but also for how middle managers communicate ratings and make employees perceive compensation systems as more effective.

### 5.2. Practical Implications

This study offers several important practical contributions relevant for managers, employees, and organizations. The findings provide guidance for improving managerial selection, leadership development, and compensation practices, particularly in settings where employee motivation and performance are critical to organizational success.

Leadership style directly shapes employee perceptions of fairness, motivation, and performance. Transformational leaders—through individualized consideration, inspirational motivation, and intellectual stimulation—establish relational contracts that build trust and strengthen psychological connections with employees. By engaging employees personally and providing coaching, feedback, and recognition, these leaders enhance intrinsic motivation and reinforce fairness in compensation and performance evaluations. Training managers in transformational competencies is therefore essential for sustaining effective compensation practices (J. K. Lee et al., 2024; Al-Rabiey et al., 2024; Song et al., 2024). The findings have direct implications for HR in recruitment, promotion, and leadership development. HR should assess transformational leadership traits when selecting or promoting managers, as such leaders foster positive employee relationships, improve compensation perceptions, and drive performance. Leadership programs should train managers in relational competencies like trust-building, active listening, and fairness. HR policies should also reward behaviors that enhance employee engagement and well-being.

At the organizational level, relational contracts add strategic value by enhancing the effectiveness of formal compensation and evaluation systems. Strong interpersonal relationships and perceived fairness increase employee motivation, engagement, and productivity (Baker et al., 2002; Levin, 2003; Chan, 2021; Darrington & Howell, 2011; Gibbons & Henderson, 2012). Organizations should integrate relational mechanisms into compensation and performance management systems to address the human and emotional aspects of work. Promoting relational contracting mitigates the limitations of purely transactional approaches and supports sustainable performance outcomes.

In contexts of organizational change, uncertainty, or instability, leadership style is crucial. Employees stay committed and productive when they trust their managers and perceive fair treatment, particularly in high-pressure environments like the banking sector (Amir, 2019; González-Cánovas et al., 2024; Grafton & Mundy, 2017).

### 5.3. Limitations

This study also entails several limitations. First, the survey methodology has potential for measurement error, given that employees were asked about their perceptions of their manager, not measuring directly the leadership style of the manager or how a manager perceives themselves as a leader. This matching, or potential mismatching, between employee perceptions and manager perception of himself/herself could be an interesting avenue for future research. Second, the SEM model exhibited lower-than-ideal values for AVE (0.229) and CR (0.607), indicating potential issues with convergent validity and internal consistency. While the construct was retained for theoretical reasons, this represents a limitation of the current model and should be addressed in future studies through refinement of the measurement scale. Third, next to leadership styles, the identity that is created at a branch or the identity with top management or the level of empowerment and delegation can be important aspects that further explain the perceptions of the use of relational contracts. Fourth, while the results suggest that transformational leadership leads to greater use of relational contracts, reverse causation is also possible. Leaders who frequently engage in trust-based, informal agreements may be perceived by employees as more transformational over time. In this case, it is not transformational behavior driving relational contracts, but rather strong relational dynamics shaping how leadership is interpreted. This reverse causality raises important questions for both researchers and HR practitioners. From a research perspective, it suggests that longitudinal or experimental designs may be necessary to disentangle the directionality of the relationship between leadership style and relational contracting. From an HR perspective, it implies that fostering a culture of trust and informal collaboration might cultivate or enhance transformational leadership perceptions, even among leaders who may not initially demonstrate strong transformational traits.

Finally, this research analyzed one bank, which had a specific compensation system for its branches, which may limit the external validity of our results.

The study focuses on a single organization—an international bank with a specific compensation system implemented across its branch network. While this setting provides rich context for analysis, it also presents limitations in terms of external validity. The uniqueness of the bank’s compensation structure may constrain the generalizability of the findings. However, the system examined is relatively common within the European banking sector and shares similarities with compensation frameworks in other industries where managers retain discretion in evaluating employee performance.

In contexts where objective performance measures are difficult to apply—such as in service-oriented roles or knowledge work—it becomes particularly important to understand how middle managers can foster trust-based environments to make relational contracts effective (Campbell, 2012; M. Gibbs et al., 2004). Therefore, while this study offers valuable insights, replication in other organizational and cultural settings is essential to confirm the broader applicability of the findings.

Future research could examine similar dynamics in different sectors, such as healthcare, education, or public administration, where relational dynamics and subjective performance evaluations also play a significant role. Moreover, while this study finds that transformational leadership yields stronger benefits than transactional leadership in this banking context, it would be valuable to explore whether and when transactional leadership offers advantages. For example, in creative industries or research and development environments, a more hands-off, transactional (or even laissez-faire) approach may not necessarily be less effective, and might better suit certain types of employees or tasks.

Despite its contextual limitations, this study contributes to a growing body of evidence suggesting that compensation system effectiveness is not only a function of formal structures but also of relational mechanisms shaped by leadership style. By highlighting the mediating role of fairness perception and intrinsic motivation, the findings offer a foundation for future cross-disciplinary and cross-cultural research into the role of relational contracts in performance management.

## Figures and Tables

**Table 1:** Descriptive statistics (N = 106).

	Mean	SD	Min	Max	Cronbach’s Alpha
**Dependent variable**					
Compensation system effectiveness	3.18	0.65	1	4.67	0.86
**Independent variables—mediators**					
Relational contracts	3.88	0.62	1.43	5.00	0.78
Transformational leadership style	4.16	0.61	2	5	0.96
Transactional leadership style	2.93	0.79	1	5	0.71
Fairness outcome	3.17	1.01	1	5	0.87
Intrinsic motivation	3.57	0.71	1.67	5	0.86
**Control variables**					
Experience	10.12	5.05	1	30	NA
Number of employees	14.22	8.13	5	29	NA
Long-term pay	3.29	0.63	1	5	0.48
Pay decentralization	2.46	0.85	1	5	0.64
Sex	0.76	0.42	0	1	NA

Sample based on n = 106 employees. Items of the model are defined in Appendix A. The average score on the items was used to calculate the measure of interest. *Experience* is an employee’s number of years of experience at the bank. *No. of employees* is the average number of employees at branch level. *Long-term pay* is a measured construct using the average of the following three items of the survey: (1) The pay system in this organization has a long-term orientation. It focuses employee attention on 2 or more years’ goals. (2) The pay system in this organization rewards employees for short-term accomplishments during a fixed time period (e.g., annual or semi-annual performance reviews). (3) Our pay policies recognize that long-term results are more important than short-term results. *Pay decentralization* measures the extent to which a branch manager has discretion on pay policies, using the average of two items of the survey. The following items were used: (1) The branch manager in each business unit has the freedom to develop its own compensation programs. (2) There is a minimum of interference from corporate headquarters with respect to pay decisions made by branch managers. *Sex* is 0 for male and 1 for female. NA indicates data not available/applicable.

**Table 2:** Pearson correlation coefficients for compensation system effectiveness attributes.

		1	2	3	4	5	6	7	8	9	10
1	Relational contracts	1									
2	Fairness outcome	0.52 **	1								
3	Intrinsic motivation	0.49 **	0.35 **	1							
4	Experience	−0.22 **	−0.07	−0.06	1						
5	Number of employees	0.05	0.02	0.27 *	−0.09	1					
6	Long term pay	0.15	0.15	0.19 *	−0.05	0.07	1				
7	Pay decentralization	0.03	0.18	0.13	0.01	0.36 **	0.12	1			
8	Sex	0.014	0.09	0.08	0.19 *	−0.07	0.17	0.08	1		
9	Transformational leadership style	0.49 **	0.29 **	0.27 **	−0.19 *	−0.20 *	0.31 **	−0.19	0.06	1	
10	Transactional leadership style	−0.07	0.04	−0.01	−0.05	0.13	0.15	0.19 *	0.08	0.04	1
11	Compensation system effectiveness	0.38 **	0.39 **	0.53 **	−0.03	0.25 **	0.35 **	0.36 **	0.06	0.13	0.21 **

* Correlation is significant at the 0.05 level (2-tailed). ** Correlation is significant at the 0.01 level (2-tailed). All items are defined in Appendix A and in Table 1.

**Table 3:** Regression of relational contracts on compensation effectiveness and mediating variables fairness outcome and intrinsic motivation.

Models	(1) Compensation System Effectiveness			(2) Fairness Outcome			(3) Intrinsic Motivation		
	Beta	t	Sig.	Beta	t	Sig.	Beta	t	Sig.
Relational contracts	0.31	3.55	0.00	0.51	5.78	0.00	0.45	5.18	0.00
Controls									
Experience	0.07	0.77	0.44	0.03	0.36	0.72	0.05	0.61	0.54
Number of employees	0.18	1.97	0.05	−0.07	−0.73	0.47	0.26	2.83	0.01
Long-term pay	0.27	3.11	0.00	0.05	0.57	0.57	0.10	1.15	0.25
Pay decentralization	0.19	2.11	0.04	0.18	1.98	0.05	0.02	0.18	0.86
Sex	−0.02	−0.25	0.81	0.04	0.42	0.67	0.05	0.60	0.55
Observations (n)		106			106			106	
R-squared		0.31			0.30			0.31	

Estimated with OLS-based regressions. R-squared denotes the unadjusted R-squared value. *p*-Values are two-tailed. All items are defined in Appendix A.

**Table 4:** Effect of relational contracts on compensation effectiveness when mediators are included.

Model	Compensation System Effectiveness		
	Beta	t	Sig.
Relational contracts	0.08	0.79	0.43
Outcome fairness	0.15	1.63	0.11
Intrinsic motivation	0.33	3.55	0.00
Controls			
Experience	0.04	0.55	0.58
Number of employees	0.10	1.16	0.25
Long term pay	0.23	2.80	0.01
Pay decentralization	0.16	1.83	0.07
Sex	−0.04	−0.55	0.58
Observations (n)		106	
R-squared		0.41	

Estimated with OLS-based regressions. R-squared denotes the unadjusted R-squared value. *p*-values are two-tailed. All items are defined in Appendix A and in Table 1.

**Table 5:** Transformational and transactional leadership styles as antecedents of relational contracts.

Models	(1) Relational Contracts			(2) Relational Contracts		
	Beta	t	Sig.	Beta	t	Sig.
Transformational leadership style	0.54	5.61	0.00			
Transactional leadership style				−0.08	−0.82	0.41
Controls						
Experience	−0.10	−1.17	0.24	−0.22	−2.23	0.03
Number of employees	0.13	1.38	0.17	0.04	0.41	0.68
Long-term pay	−0.05	−0.56	0.57	0.13	1.34	0.18
Pay decentralization	0.14	1.44	0.15	0.03	0.25	0.81
Sex	−0.01	−0.12	0.90	0.03	0.34	0.73
Observations (n)		106			106	
R-squared		0.29			0.76	

Estimated with OLS-based regressions. R-squared denotes the unadjusted R-squared value. *p*-Values are two-tailed. All items are defined in Appendix A.

**Table 6:** Effects of ratings on performance outcomes for different levels of compensation effectiveness the first time they are cited.

Panel A: Effect of ratings on performance outcomes (i.e., non-working hours) and impact of compensation effectiveness						
Models	(1) Non-working hours			(2) Non-working hours		
	Beta	t	Sig.	Beta	t	Sig.
Ratings	−0.18	−1.66	0.10	0.75	1.44	0.15
Compensation system effectiveness				0.71	1.56	0.12
Compensation system effectiveness × Ratings				−1.21	−1.81	0.07
Controls						
Experience	0.09	0.88	0.38	0.76	0.70	0.48
Number of employees	0.09	0.82	0.41	0.09	0.83	0.40
Long-term pay	0.14	1.31	0.19	0.09	0.74	0.46
Pay decentralization	0.24	2.11	0.04	0.24	2.11	0.04
Sex	0.07	0.64	0.52	0.09	0.84	0.40
Observations (n)		83			83	
R-squared		18.9%			22.9%	
Panel B: Effects of ratings on performance outcomes (i.e., non-working hours) for low and high perceived compensation system effectiveness						
	Below the median value			Above the median value		
Models	(1) Non-working hours			(2) Non-working hours		
	Beta	t	Sig.	Beta	t	Sig.
Ratings	0.07	0.35	0.73	−0.23	−1.79	0.08
Controls						
Experience	0.60	2.76	0.01	0.04	0.28	0.78
Number of employees	0.47	2.23	0.04	0.07	0.47	0.64
Long-term pay	0.18	0.83	0.42	0.09	0.72	0.47
Pay decentralization	0.27	1.39	0.19	0.24	1.71	0.09
Sex	0.03	0.14	0.89	0.12	0.91	0.36
Observations (n)		22			61	
R-squared		43.4%			20.5%	

Estimated with OLS-based regressions. R-squared denotes the unadjusted R-squared value. *p*-Values are two-tailed. Sample is based on n = 83 employees due to data limitations on the dependent variable non-working hours and the ratings (that are missing for some employees). Variables of the model are defined in Appendix A. In addition, the number of non-working hours in 2016 was used as a proxy for employees’ performance outcome. Ratings are given by branch managers, and they can range from 1 to 3 (1 stands for below the expectations; 2 stands for average performance; and 3 stands for above the expectations). Higher ratings thus represent higher performance. Panel A presents the sample of n = 83. Panel B provides a split analysis for compensation effectiveness. A significant number of observations (n = 23) are at the median of 3 on compensation effectiveness. These observations are classified into the high compensation effectiveness group.

## Data Availability

Data available from the authors upon request.

## Notes

1 This paper employes the definitions of transactional and transformational leadership styles as per McDermott et al. (2013). Specifically, transactional leaders often use particular, measurable goals, coordinating the work and monitoring accomplishments and deliverables of their team, while transformational leaders operate by inspiring people, linking results to values and fostering common goals and mutual identification.

2 These systems are used in many organizations and are often referred to as forced rankings, forced distributions, or rank-and-yank systems where people are ranked on a bell curve or other pre-defined distribution.

3 Employee salary comprises two parts: a basic salary and a bonus. Managers suggest the allocation of the bonus pool between employees; the decision is based on employee performance. The basic salary is pre-defined for all positions in the bank, and managers have no influence on that part of the compensation.

4 All employees gave their written consent before the survey, stressing the purpose of the study and confidentially of their participation. We emphasized anonymity to top management, employees, and branch managers to assure them that answers of employees would be treated with confidentiality. The intention was to obtain answers that reflect the genuine opinion of the employee. To further promote the survey among employees, we offered the top management team to discuss anonymized results in an anonymized report, once the study was completed.

5 We run an independent-sample *t*-test in order to compare the responses from branches we visit early versus responses from branches we visit late (Lindner et al., 2001). In order to do so, we split the dataset into two groups. The first group contains the first 25% of the responses, and the second contains the 25% of the responses collected at the end. Results show that there is not a statistically significant difference in the mean compensation system effectiveness scores for early and late responses [t(56) = −1.102, *p* = 0.28].

## Appendix A

**Table A1:** Scales for the theoretical constructs and their respective items.

Scale	Cronbach Alpha	Components
A Relational contracts	0.78	A1. I expect to gain promotion in this company with length of service and effort to achieve goals. A2. I expect to grow in this organization. A3. I feel part of a team in this organization A4. I feel this company reciprocates the effort put in by its employees. A5. I have a reasonable chance of promotion if I work hard. A6. I will work for this company indefinitely. A7. I am heavily involved in my place of work.
B Fairness outcome	0.87	B1. I think that rewards are relative to effort. B2. I think that rewards are relative to responsibilities. B3. I think that rewards are relative to job stress. B4. I think that rewards are relative to education and training.
C Intrinsic motivation	0.86	C1. The tasks that I do at work themselves represent a driving power in my job. C2. The tasks that I do at work are enjoyable. C3. My job is meaningful. C4. My job is very exciting. C5. My job is so interesting that it is a motivation in itself. C6. Sometimes I become so inspired by my job that I almost forget everything else around me.
D Compensation system effectiveness	0.86	D1. Our pay policies and practices are highly effective. D2. Management is satisfied with the way the compensation system contributes to the achievement of overall organizational goals. D3. Our pay policies and practices appear to enjoy widespread acceptability among employees. D4. Our pay policies and practices greatly contribute to the retention of employees. D5. Our pay policies and practices greatly contribute to the attraction of employees. D6. Our pay policies and practices greatly contribute to the motivation of employees.
E Multifactor Leadership Questionnaire	0.92	E1. My branch manager makes others feel good to be around him/her. E2. My branch manager expresses with a few simple words what we could and should do. E3. My branch manager enables others to think about old problems in new ways. E4. My branch manager helps others develop themselves. E5. My branch manager tells others what to do if they want to be rewarded for their work. E6. My branch manager is satisfied when others meet agreed-upon standards. E7. My branch manager is content to let others continue working in the same ways always. E8. Others from the branch have complete faith in our branch manager. E9. My branch manager provides appealing images about what we can do. E10. My branch manager provides others with new ways of looking at puzzling things. E11. My branch manager lets others know how he/she thinks they are doing. E12. My branch manager provides recognition/rewards when others reach their goals. E13. As long as things are working, my branch manager does not try to change anything. E14. Whatever others want to do is OK with my branch manager. E15. Others are proud to be associated with my branch manager. E16. My branch manager helps others find meaning in their work. E17. My branch manager gets others to rethink ideas that they had never questioned before. E18. My branch manager gives personal attention to others who seem rejected. E19. My branch manager calls attention to what others can get for what they accomplish. E20. My branch manager tells others the standards they have to know to carry out their work. E21. My branch manager asks no more of others than what is absolutely essential.

**Table A2:** Demographic and Organizational Characteristics of the Sample.

Variable	Category/Description
Sex	Female (81)
	Male (25)
Age	Mean = 37.50 (SD = 6.70)
Work Experience	Mean = 10.12 years (SD = 5.05)
Branch Size	Mean = 14.2 employees (SD = 8.13)
Number of Branches	24 branches across 5 major cities in Serbia
Main Location	Majority in Belgrade; balanced across municipalities
Eligibility Criteria	≥5 employees per branch, ≥1 year in current position
Response Rate	106 usable responses out of 206 distributed
